# Pathways to Low Fertility: 50 Years of Limitation, Curtailment, and Postponement of Childbearing

**DOI:** 10.1007/s13524-019-00848-5

**Published:** 2020-01-22

**Authors:** Ian M. Timæus, Tom A. Moultrie

**Affiliations:** 1 Department of Population Health, London School of Hygiene & Tropical Medicine, London, UK; 2 Centre for Actuarial Research, University of Cape Town, Cape Town, South Africa

**Keywords:** Fertility transition ∙ Parity progression ∙ Birth intervals ∙ Birth control ∙ Postponement

## Abstract

This study applies survival analysis to the birth histories from 317 national surveys to model pathways to low fertility in 83 less-developed countries between 1965 and 2014. It presents period measures of parity progression, the length of birth intervals and total fertility that have been standardized fully for age, parity, and interval duration. It also examines parity-specific trends in the proportion of women who want no more children. Outside sub-Saharan Africa, fertility transition was dominated by parity-specific family size limitation. As the transition progressed, women also began to postpone their next birth for lengthy periods in many countries. During the first half of the fertility transition in much of sub-Saharan Africa and in some other countries, however, women stopped childbearing without targeting particular family sizes. Moreover, birth intervals in sub-Saharan Africa have been lengthening since the onset of the transition. Birth control is not restricted to a dichotomy between limitation and spacing. Other reasons for curtailing childbearing and postponing having another birth also shape countries’ pathways through fertility transition.

**Electronic supplementary material:**

The online version of this article (10.1007/s13524-019-00848-5) contains supplementary material, which is available to authorized users.

## Introduction

Much of the demographic literature on fertility transition and fertility intentions is phrased in terms of limitation and spacing, which are viewed as contrasting modes of birth control that reflect women’s preferences about the size of their families and the intervals between their births, respectively (e.g., Bongaarts 1992; Bradley et al. 2012; Knodel 1987; Okun 1995; Van Bavel 2004; van Poppel et al. 2012; Westoff and Koffman 2010). In this article, we deploy evidence from across the Global South to argue that this paradigm provides an oversimplified view of the reasons why women control their fertility and fails to adequately describe or explain the diverse pathways to low fertility that countries follow.

Caldwell et al. (1992) were the first researchers to suggest that for historical and cultural reasons, Africa would undergo a unique fertility transition that was less concentrated among older, high-parity women than elsewhere. The evidence for and against this assertion has been debated ever since (e.g., Bongaarts 2017; Bongaarts and Casterline 2013; Brass et al. 1997; Moultrie et al. 2012). Moreover, recent research has found that very long birth intervals have become common during the course of fertility transition in many countries. This was documented first for South Africa (Timæus and Moultrie 2008), then in other parts of sub-Saharan Africa (Moultrie et al. 2012), and subsequently in many other parts of the world (Casterline and Odden 2016). This development is difficult to interpret in terms of spacing as it is usually conceptualized. Median closed birth intervals of 42 or more months cannot plausibly result from the desire to avoid pregnancy until the mother’s previous birth has reached a certain age or developmental milestone (e.g., the child being weaned or starting to walk). Instead, they reflect postponement of births, which can be conceptualized as a third motivation for birth control, distinct from both limitation and spacing (Timæus and Moultrie 2008). Postponers have decided neither that they have enough surviving children nor that they want another child once their youngest child is old enough. Rather, they are deferring the decision as to whether or when to have more children; they have decided only that they want no more for the moment.

Demographic analyses that partition contraceptive use and its ensuing impact on aggregate fertility patterns into limitation and spacing have assumed that women’s reproductive histories (the number, ages, and survival of their children) are as central to their fertility decision-making as they are to the research agenda of demography. Other factors that might affect these decisions are conceptualized either as operating via women’s preferences for a particular family size or interval length, or as idiosyncratic and unimportant. However, women may also seek to avoid childbearing for reasons that are unrelated to their reproductive histories, such as difficulties in their relationship with their husband or partner, perceived economic hardship or insecurity, or ill health. Such concerns may lead women to avoid childbearing even if they would want another birth in more favorable circumstances. Postponing having another birth for such reasons has different demographic consequences from the spacing of births until the previous child reaches a particular age. Spacing of the latter type is inherently self-limiting—that is, once the preferred interval has been achieved, the motivation to avoid conception disappears—and can have only a moderate impact on overall fertility. In contrast, postponement of the next birth for nondemographic reasons can continue indefinitely and may have a major impact on overall fertility (Timæus and Moultrie 2013).

Women who postpone their next birth may end up not having another child because they have become infecund. Such women have been described as permanent (Lightbourne 1985) or perpetual postponers. Equally, women may respond to the kind of personal and socioeconomic circumstances that motivate postponement by deciding instead to stop childbearing. They may do so either after postponing having a baby for a period or at the outset of a birth interval. We refer to this form of stopping, which occurs for reasons unrelated to women’s parity, as the “curtailment” of childbearing.^1^ Demographic theory has seldom focused on women who stop children bearing for nondemographic reasons, yet curtailment differs conceptually and analytically from limitation of one’s family to a specific preferred size. As this article demonstrates, curtailment also has different implications for aggregate fertility outcomes.

This study investigates pathways to low fertility since the 1960s in as many countries as possible. We use a standardized analytical framework to assess the contributions that limitation, curtailment, spacing, and postponement have made to fertility decline across the globe. Our work adds to the empirical findings of previous research on this topic in several ways. First, following Towriss and Timæus (2018), we examine trends in parity progression as well as in the length of birth intervals. Second, unlike Moultrie et al. (2012), we look at all the world regions that have been undergoing fertility transition during the last 50 years. Third, unlike Casterline and Odden (2016), we examine not only the first-to-second birth interval but all birth intervals and parity-age-duration–adjusted total fertility.

## Data and Methods

Our analysis uses individual-level data to produce national-level estimates of birth interval dynamics and fully standardized measures of total fertility. We deploy 317 World Fertility Survey (WFS) surveys, Demographic and Health Surveys (DHS), and Reproductive Health Surveys (RHS) from 83 countries to investigate trends and patterns in family formation over the course of their fertility transitions. The WFS was conducted in the late 1970s and early 1980s. It collected full birth histories, allowing investigation of fertility during the previous 15 years. The analysis therefore covers the period from 1965 to 2014. The database of demographic surveys includes every DHS in the public domain in early 2019 and all the RHS that collected birth histories. It excludes nine WFS surveys conducted before 1985 in countries that have not undertaken a RHS or DHS since.

Almost one-half the surveys were conducted in sub-Saharan Africa, but the database includes surveys from most parts of the world other than its high-income countries (see Table 1; a full list of the surveys included in the analysis is provided in Table A1 of the online appendix). It does not include China. Not only is this database incomplete in its geographical coverage, but it captures only portions—of varying duration—of the entire fertility transition in each country. In 12 countries, the most recent survey took place before 2000. In several of these countries, such as Mexico, Tunisia, and Sudan, this means that the surveys document only the initial stages of their fertility transition. In others, such as Brazil and Sri Lanka, total fertility was close to or less than three children per woman by the time the last survey was conducted. These limitations of the empirical record are inescapable; however, we try to avoid misleading comparisons when presenting the results and to draw the reader’s attention to such complexities during the discussion of their interpretation.

**Table 1:** Fertility surveys included in the analysis according to date of fieldwork and region

	Period									
Region and Subregion	1975–1979	1980–1984	1985–1989	1990–1994	1995–1999	2000–2004	2005–2009	2010–2014	2015–2019	Total
Sub-Saharan Africa	5	4	9	16	22	21	22	34	11	144
East	1	1	4	7	9	10	7	12	6	57
Middle	1	0	0	2	2	3	3	5	1	17
Southern	1	0	0	1	1	2	3	2	1	11
West	2	3	5	6	10	6	9	15	3	59
Latin America and Caribbean	10	1	9	10	13	12	11	8	2	76
Caribbean	3	1	2	2	2	2	2	2	1	17
Central America	2	0	3	2	4	4	4	2	0	21
South America	5	0	4	6	7	6	5	4	1	38
Middle East and North Africa	5	2	4	5	3	4	3	3	0	29
East Europe and Central Asia	0	0	0	0	4	1	5	3	3	16
South and Southeast Asia	7	0	3	6	7	6	9	8	6	52
South Asia	4	0	1	3	4	2	5	4	3	26
Southeast Asia	3	0	2	3	3	4	4	4	3	26
Total	27	7	25	37	49	44	50	56	22	317

We have argued previously that stopping, spacing, and postponement—defined in a formal but intuitive way—have different and unambiguous effects on changes in the interval duration-specific fertility schedule (Timæus and Moultrie 2013). The same is true of curtailment: although parity-specific limitation of family size is concentrated on specific preferred small- or medium-sized families, the incidence of curtailment and perpetual postponement is unrelated to, or increases with, parity. Thus, the analysis focuses on parity-specific measures of the proportion of women who progress to the next birth and summary indicators of trends in the shape of interval duration–specific fertility distributions.

**Fig. 1:**
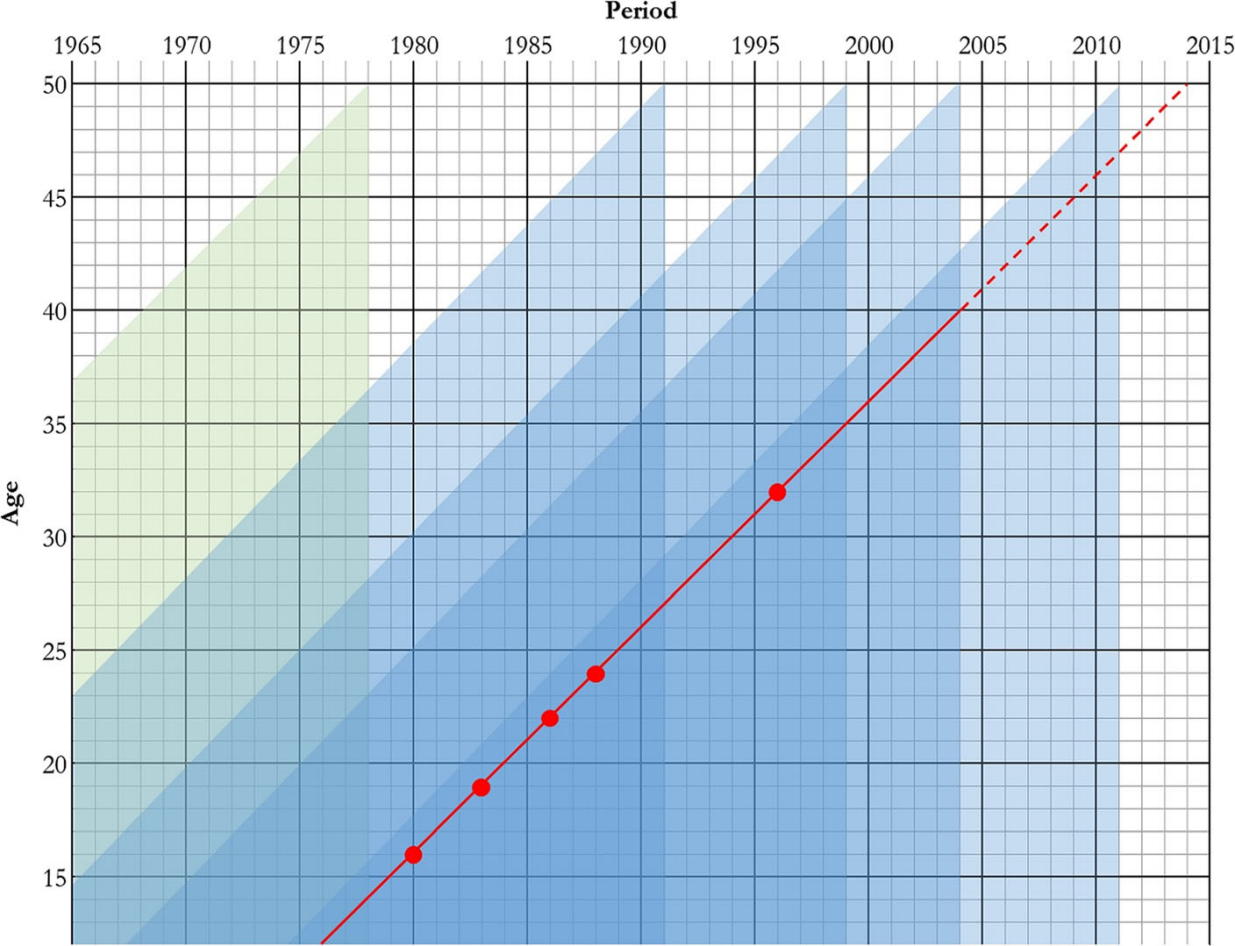
Lexis representation of the data analysis

All the survey data from a single country are combined, preserving the information on the design of each survey. Each woman’s birth history is split into episodes defined by quinquennial period and the interval since her previous birth (splitting at durations 9, 18, 24, . . . , 72, 84, . . . , 144 months), and her parity and five-year age group at the start of each episode are calculated. Figure 1 illustrates this for a country—modelled loosely on Cameroon—that has conducted a WFS study and four DHS. All the births in the birth histories reported by women in the WFS study occurred within the shaded area at the left of the figure. The other four shaded areas represent the ages and periods covered by the different sets of DHS birth histories. For example, all four DHS provide information on the births of women who were younger than 25 in the period 1985–1990, but only the most recent survey obtained reports on 2005–2011. To produce a full set of estimates for women aged 40 or older, one has to extrapolate across the unshaded areas of the diagram based on reporting about the shaded areas.

We conduct a period-based analysis of parity progression and interval dynamics (Ní Bhrolcháin 1987). In other words, all the fertility rates are based on births and exposure to women in a specific five-year age group, during a specific five-year period, represented by one of the larger black boxes shown in Fig. 1. However, the primary time dimension of interest is interval duration–specific fertility. This is illustrated by a diagonal line representing the life history of a woman who was born at the end of 1964 and was interviewed in the survey conducted at the end of 2004. Thus, her information is censored at this point, and the rest of her reproductive life is represented by a dotted line.

First births are modelled by age. Thus, the woman’s exposure between age 12 and her first birth at age 16, indicated by a solid circle on her timeline in Fig. 1, counts in the denominators of the rates for the first two age groups during 1975–1980. She then contributed exposure to the second birth interval at durations of 0–3 years, in the period 1980–1985, ending when she had her second birth at age 19. Following this birth, she had three additional children—at intervals of 3, 2, and 8 years—followed by 8 years of exposure in the open interval that began with the birth of her fifth child and extended to the date when she was interviewed.

The methods used for the analysis both build on our earlier research and resemble the approach Retherford et al. (2010, 2013) proposed. We use survival analysis—specifically, a Poisson model with an exposure offset—to model the trend in the log age-order-duration–specific birth rates in each country. Detailed accounts of this model are available elsewhere (Moultrie et al. 2012; Towriss and Timæus 2018). The key features of the model are as follows:

- The main effects of parity, age, interval duration, and period are modelled using dummy variables rather than by imposing a specific functional form on the data.
- Differential change in fertility by parity and interval duration is modelled using continuous variables and their interactions to average across random fluctuations in the data and enable interpolation between surveys to estimate a complete set of fertility rates for older women.
- The age dummy variables pick up the biological decline in fecundity with age; they are not interacted with other variables, including time, to avoid overfitting the data.

This model differs from that used by Retherford and his coauthors (1) in this last regard; (2) as it estimates fertility by five-year period from a data set including all available surveys rather than for a five-year period before each survey; and (3) as fertility at all parities is estimated from a single model including interactions with parity. Each of these differences serves to increase the robustness of the estimates. First births are modelled separately as a function of quinquennial period and women’s age in years using a similar, but simpler, Poisson regression model.

The output from the regression model is a complete series of fitted age-order-duration–specific fertility rates for each five-year period covered by the fertility survey data. From these, one can construct summary life table estimates of fertility by birth order. Using the same methods as Retherford et al. (2013), which were described first by Rallu and Toulemon (1994), we construct a multistate life table model of the family-building process. This calculates synthetic cohort indices of the final fertility outcomes that a cohort of pubertal girls would experience if they went through life experiencing the age-order-duration–specific rates of the period in question. This analysis yields a complete set of parity progression ratios (PPRs) for each period, together with period estimates of the median duration of closed birth intervals of each order and of all orders. Moreover, by summing the synthetic cohort estimates of births of different orders, one can calculate a fully parity-age-duration–adjusted index of total fertility (Rallu and Toulemon proposed the acronym PADTFR) analogous to the conventional age-adjusted total fertility rate. This procedure avoids the issue of how to weight births at each parity to calculate overall fertility by generating the population at risk of giving birth according to parity from the contemporaneous fertility rates for lower birth orders.

The analysis proceeds by using several heuristic diagnostics calculated from the life tables for duration-specific fertility according to parity to assess the features of the fertility transition in each country. Timæus and Moultrie (2008) suggested that one can use the coefficients of the regression model used to smooth the rates to test for the statistical significance of changes and differences in fertility patterns. Experience suggests, however, that not only are such indicators difficult to interpret, but the approach suffers from the limitation that even minor changes in fertility are usually statistically significant given the large samples of births available for most countries.

The first two diagnostics are based on the series of period PPRs for each quinquennium. These PPRs measure the proportion of women of each parity who have another child within 12 years. One can infer that parity-specific stopping occurs if plots of progression across parities become increasingly concave over time (Brass and Juárez 1983; Brass et al. 1997). This occurs when progression to the third birth, and usually also the fourth birth, drops by more than progression to higher-order births, dragging the curve downward. In contrast, PPRs that decrease linearly with increasing parity would suggest that women have begun to reduce their family sizes without having developed clearly defined desired family sizes. The relative size of the reduction in progression at smaller and larger family sizes is summarized by comparing progression to the fourth birth (calculated by multiplying together the first four PPRs) with progression from the fourth to the eighth birth (calculated by multiplying together the next four ratios). In addition, the shape of the most recent parity distribution is summarized by comparing the ratio of the differences between PPR_0_ and PPR_4_ on the one hand, and PPR_4_ and PPR_8_ on the other, where PPR_*n*_ denotes the proportion progressing from the *n*th to the *n* + 1th birth.

The next diagnostic captures the trend in the median length of closed birth intervals. This indicator is also calculated on a period basis. It is simply the duration since the previous birth by which one-half of the women who progress to another birth within 12 years have done so, given the fertility rates of the period.

Spacing and postponement produce different patterns of change in the schedule of duration-specific hazard rates (Timæus and Moultrie 2013). Birth spacing is identified by determining whether the life table probability of closing a birth interval at less than 30 months (*B*(30)) had dropped by more over time than the probability of closing the birth interval at 30–60 months (_30_*b*_*3*0_).^2^ Postponement raises the proportion of birth intervals that are closed at long durations, resulting in a counterclockwise rotation of the duration-specific fertility schedule. Specifically, it is identified by examining how much the life table probability of closing a birth interval at 60–120 months (_60_*b*_*6*0_) in a country dropped compared with the probability of closing the interval within five years (*B*(60)). We classify countries in terms of each of the indices by estimating the regression of the first measure on the second and calculating the residuals. Countries in which both residuals are small or the median closed birth interval rose by less than three months are classified as neither spacing nor postponing by contraceptive means.^3^ We subdivide the countries characterized by postponement into two groups. Countries in which the median interval increased by more than 12 months during the period of observation or rose to 48 or more months are described as experiencing substantial postponement. Countries with a combination of an increase in the median interval of at least 6 months, a final median of more than 42 months, and a relative rise in fertility at long interval durations are also placed in this group.

The pattern of fertility decline in each of the 83 countries is classified according to these measures; the results are assessed to examine diversity and regional clustering in the pathways by which different countries have progressed through the fertility transition and to identify countries that have undergone exceptional transitions. To avoid producing overly cluttered figures, we present results for alternate quinquennia, terminating in 2005–2009, together with estimates for 2010–2014 in countries for which they are available.

Last, we examine the trend across successive surveys in the proportion of currently married women by parity who report that they want no more children in order to assess whether the evolution of women’s fertility preferences mirrors trends in parity progression. The measure is calculated for reportedly fertile married women who either gave birth in the previous year or were yet to start childbearing.^4^

## Results

**Fig. 2:**
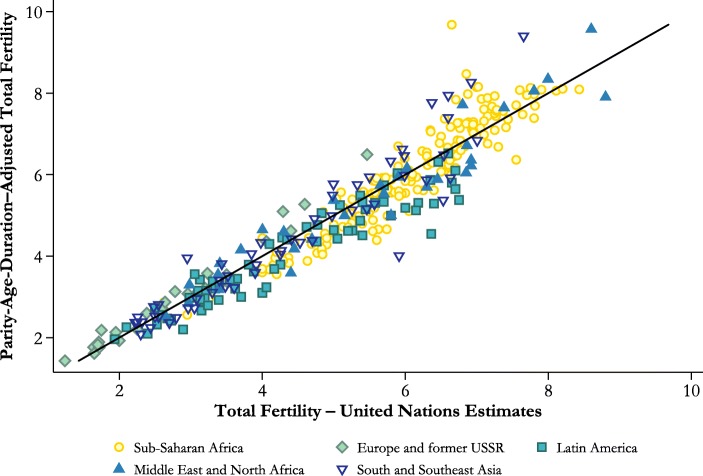
Comparison of estimated parity-age-duration–adjusted total fertility with the United Nations estimates of total fertility for the same quinquennium, 344 estimates for 83 countries

Figure 2 compares our estimates of parity-age-duration–adjusted total fertility with estimates for the same periods published by the United Nations in *World Population Prospects* (UN Population Division 2017). The two series agree closely, although the United Nations estimates tend to be slightly lower in populations with very high fertility and slightly higher in populations in which total fertility is between four and six children per woman. One would not expect the two series to be identical, not only because the United Nations estimates standardize only for age, not parity and interval duration, but also because they were made using sources other than fertility surveys. Nevertheless, the close agreement between the series represents external evidence of the validity of our model of parity-age-duration–specific fertility. The remainder of the analysis excludes the indices for Benin and El Salvador in 1965–1969, which yielded implausible estimates of the PADTFR, probably as a result of errors in women’s reports about a period several years before the earliest survey in each country.

**Fig. 3:**
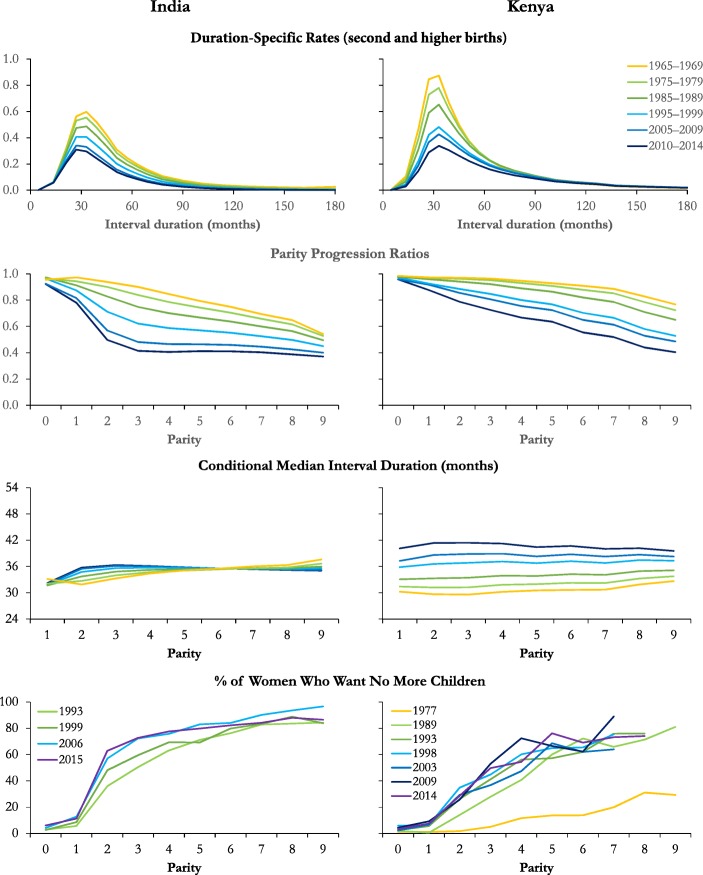
Trends in (1) fertility by interval duration, (2) progression to the next birth, (3) the median duration of closed intervals by parity in successive quinquennia, and (4) the percentage wanting no more children among married women who gave birth in the previous year or are childless by parity in successive surveys, India and Kenya

Because it is infeasible to present and discuss detailed results for 83 countries, Fig. 3 presents illustrative results for two: India and Kenya.^5^ India exemplifies a transition to low fertility that has been driven by parity-specific birth control. Fertility fell at all interval durations but by more at long durations than short ones. Although all the period PPRs dropped, the proportion of women progressing to a third and fourth birth fell the most. Thus, the plot of the period PPRs by parity are initially linear but become strongly concave over the 45-year period examined. The period median closed birth interval is about 34 months, varying only slightly by parity. Moreover, apart from having increased by two to three months last century among women progressing to their third and fourth births, it has changed little over time. The plots of the proportions of women by parity who want no more children are a mirror image of the PPRs: they are highly convex, rising dramatically for women who have had exactly two children, compared with those who just have one.^6^

Fertility transition followed a different path in Kenya. Fertility declined far more at interval durations of less than five years than at longer durations, producing the flattening of the duration-specific fertility distribution that characterizes postponement. According to these fully standardized estimates, fertility decline slowed at the beginning of the twenty-first century but did not experience a reversal. As in India, the period median length of birth intervals varied little by parity. However, it has been increasing steadily since fertility began to fall. By 2010–2014, it was 41 months. Notably, the decline in the PPRs with increasing parity in Kenya remained close to linear even though the PPRs dropped substantially. It also became steeper: progression from the fourth to the eighth birth dropped by more than progression to the fourth birth. Thus, the more children that Kenyan women had, the more likely they were not to have another birth. Comparatively, in India by 2015, the proportion of women progressing varied very little with parity after the first three births.

To some extent, Kenyan women may have been prevented from progressing as intervals lengthened by the decline in their fecundity with age (i.e., the number of perpetual postponers may have risen). The drop in the higher-order progression ratios was so large, though, that it could be due only to the widespread use of birth control to stop childbearing. However, women cease childbearing at a wide range of family sizes. No evidence exists that their final outcomes converged on small desired family sizes. Mirroring this, the proportion of the women who want no more children rose steadily with parity rather than rising sharply after they had two children, as in India.

**Fig. 4:**
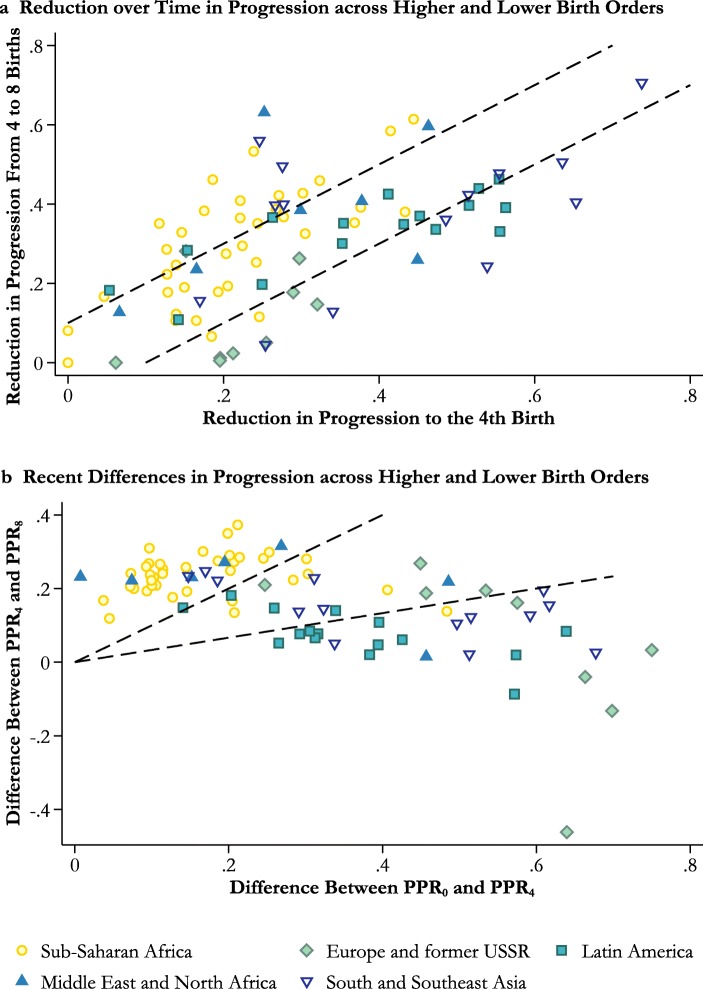
Relationship between higher-order and lower-order parity progression in 83 countries

Panel a of Fig. 4 compares period probabilities of progression from the fourth to the eighth birth with those to the fourth birth in all 83 countries. The dashed lines enclose the set of countries in which the absolute difference between the measures is less than 0.1. Like Kenya, nearly all the other African countries experienced a decline in progression from the fourth to the eighth birth that was at least as large as the decline in progression to the fourth birth. The two exceptions are South Africa, where fertility was already fairly low, and The Gambia. Moreover, inspection of the detailed results for sub-Saharan Africa included in the online appendix (Fig. A1) reveals that for most countries, they resemble those for Kenya: they are characterized by linear declines in progression with parity that steepened over time. Note that all the countries in panel a (Fig. 4), apart from those in the lower-left corner, experienced large declines in period parity progression. In most of Africa, however, these have not taken the form of parity-specific family size limitation.

Most countries outside mainland sub-Saharan Africa exhibit the opposite pattern of change, with larger reductions in the period probability of progressing to the fourth birth than in that of progressing from the fourth to eighth birth. A few non-African countries, however, followed an “African” pattern of decline in parity progression, with relatively large reductions among higher-parity women and little evidence of parity-specific limitation. These countries include Afghanistan, Pakistan, Jordan, Yemen, Ecuador, and several countries in which the most recent surveys date back to the 1990s (i.e., Costa Rica, Mexico, Thailand, and Uzbekistan; see Fig. A1 in the online appendix). Table 2 lists whether each of the 83 countries has a larger, smaller, or similar drop in progression from the fourth to the eighth birth than progression to the fourth birth. It also presents additional series of summary measures of the pattern of fertility change in each country. We describe these indices with reference to Figs. 4 (panel b), 5, and 6.

**Table 2:** Characteristics of the fertility transition in 83 countries

	Pattern of Stopping Childbearing			Changes in the Distribution of Birth Intervals			
Country	Most Recent TFR	Drop in PPR_4–8_ Compared With PPR_0–4_	Final Ratio of (PPR_0_–PPR_4_) to (PPR_4_–PPR_8_)	Increase in Median Closed Interval (months)	Most recent Median Closed Interval (months)	Ratio of Change in _60_*b*_60_ to Change in *B*(60)	Ratio of Change in *B*(30) to Change in _30_*b*_30_
East Africa							
Burundi	5.5	Larger	Convex	3–6	<42	Rises	Rises
Comoros	4.3	Larger	Convex	3–6	<42	Drops	Drops
Ethiopia	3.9	Similar	Convex	<3	<42	Drops	Rises
Kenya	3.7	Larger	Concave	6–12	<42	Constant	Drops
Madagascar	4.2	Similar	Concave	6–12	<42	Constant	Drops
Malawi	4.6	Larger	Convex	6–12	42–48	Rises	Drops
Mozambique	5.7	Similar	Convex	3–6	<42	Rises	Drops
Rwanda	3.6	Larger	Concave	6–12	<42	Constant	Drops
Tanzania	5.0	Similar	Convex	3–6	<42	Rises	Rises
Uganda	5.5	Larger	Convex	3–6	<42	Rises	Rises
Zambia	4.7	Larger	Convex	6–12	<42	Rises	Drops
Zimbabwe	4.4	Larger	Convex	12+	48+	Rises	Drops
Middle Africa							
Angola	6.0	Similar	Convex	<3	<42	Drops	Rises
Cameroon	4.8	Similar	Convex	3-6	<42	Rises	Rises
Central African Republic	6.7	Similar	Convex	<3	<42	Constant	Rises
Chad	6.2	Larger	Convex	<3	<42	Rises	Rises
Congo (Democratic Republic)	5.6	Similar	Convex	<3	<42	Drops	Rises
Congo (Republic)	4.7	Similar	Convex	<3	42–48	Constant	Rises
Gabon	3.8	Similar	Concave	6–12	<42	Constant	Rises
São Tomé and Principe	4.4	Similar	Convex	<3	42–48	Constant	Rises
Southern Africa							
Lesotho	3.0	Similar	Concave	12+	48+	Rises	Rises
Namibia	3.3	Similar	Concave	12+	48+	Constant	Drops
South Africa	2.8	Smaller	Very concave	12+	48+	Constant	Rises
Swaziland	4.3	Similar	Convex	3–6	<42	Constant	Rises
West Africa							
Benin	5.1	Larger	Convex	6–12	<42	Constant	Rises
Burkina Faso	5.8	Larger	Convex	3–6	<42	Constant	Drops
Côte d’Ivoire	4.8	Larger	Convex	6–12	<42	Rises	Drops
The Gambia	4.7	Smaller	Convex	<3	<42	Drops	Rises
Ghana	4.0	Larger	Convex	6-12	42–48	Constant	Rises
Guinea	5.5	Similar	Convex	<3	<42	Constant	Rises
Liberia	4.1	Larger	Convex	12+	42–48	Rises	Drops
Mali	6.7	Larger	Convex	3–6	<42	Rises	Drops
Niger	7.7	Similar	Convex	<3	<42	Rises	Rises
Nigeria	4.7	Similar	Convex	<3	<42	Constant	Rises
Senegal	4.7	Larger	Convex	3–6	<42	Rises	Rises
Sierra Leone	4.0	Similar	Convex	<3	<42	Drops	Rises
Togo	4.3	Larger	Convex	3–6	<42	Constant	Rises
Eastern Europe and Former USSR							
Albania	1.9	Smaller	Very concave	<3	42–48	Drops	Rises
Armenia	1.6	Smaller	Very concave	6–12	<42	Drops	Rises
Azerbaijan	2.2	Smaller	Very concave	<3	<42	Drops	Rises
Kazakhstan	1.9	Smaller	Concave	<3	<42	Drops	Rises
Kyrgyz Republic	3.1	Similar	Concave	6-12	<42	Constant	Drops
Moldova	1.8	Smaller	Very concave	3-6	48+	Drops	Rises
Tajikistan	3.4	Smaller	Concave	<3	<42	Drops	Rises
Ukraine	1.4	Similar	Very concave	3-6	48+	Drops	Rises
Uzbekistan	4.6	Larger	Concave	<3	<42	Drops	Rises
Caribbean							
Dominican Republic	2.3	Similar	Very concave	12+	42–48	Drops	Large drop
Haiti	2.9	Similar	Concave	6–12	42–48	Rises	Drops
Trinidad and Tobago	2.8	Similar	Concave	6–12	<42	Rises	Drops
Central America							
Costa Rica	3.4	Larger	Concave	12+	42-48	Rises	Large drop
El Salvador	2.1	Smaller	Very concave	12+	42-48	Drops	Drops
Guatemala	3.0	Similar	Very concave	6–12	<42	Drops	Drops
Honduras	2.7	Similar	Very concave	12+	42-48	Drops	Drops
Mexico	4.5	Larger	Convex	3–6	<42	Rises	Drops
Nicaragua	3.3	Similar	Very concave	6–12	<42	Drops	Drops
South America							
Bolivia	2.9	Similar	Very concave	6–12	<42	Drops	Drops
Brazil	3.1	Similar	Very concave	6–12	<42	Constant	Large drop
Colombia	2.0	Smaller	Very concave	12+	48+	Drops	Large drop
Ecuador	3.2	Larger	Very concave	12+	<42	Constant	Drops
Guyana	2.4	Smaller	Very concave	12+	42-48	Constant	Large drop
Paraguay	2.2	Similar	Very concave	12+	48+	Constant	Drops
Peru	2.6	Smaller	Very concave	12+	48+	Constant	Large drop
Middle East and North Africa (MENA)							
Egypt	3.3	Similar	Concave	6–12	<42	Constant	Large drop
Jordan	4.2	Larger	Convex	6–12	<42	Rises	Large drop
Morocco	2.8	Similar	Convex	12+	<42	Rises	Large drop
Sudan (North)	5.7	Similar	Convex	3–6	<42	Constant	Large drop
Tunisia	4.6	Similar	Convex	3–6	<42	Drops	Drops
Turkey	2.5	Smaller	Very concave	6–12	<42	Drops	Drops
Yemen	3.6	Larger	Convex	6–12	<42	Drops	Large drop
South Asia							
Afghanistan	5.1	Larger	Convex	<3	<42	Drops	Rises
Bangladesh	2.4	Similar	Very concave	12+	48+	Drops	Drops
India	2.2	Smaller	Very concave	<3	<42	Drops	Rises
Maldives	2.5	Smaller	Very concave	6–12	48+	Drops	Rises
Nepal	2.4	Smaller	Very concave	3–6	<42	Drops	Rises
Pakistan	4.3	Larger	Convex	<3	<42	Drops	Rises
Sri Lanka	3.2	Similar	Concave	3–6	<42	Constant	Rises
Southeast Asia							
Cambodia	2.4	Similar	Very concave	6–12	42–48	Drops	Rises
Indonesia	2.5	Smaller	Very concave	12+	48+	Rises	Drops
Myanmar	2.1	Smaller	Concave	<3	48+	Drops	Rises
Philippines	2.7	Similar	Very concave	6–12	<42	Constant	Drops
Thailand	3.8	Larger	Concave	6–12	<42	Constant	Drops
Timor-Leste	4.0	Larger	Convex	<3	<42	Drops	Rises
Vietnam	2.4	Smaller	Very concave	6–12	42–48	Drops	Rises

**Fig. 5:**
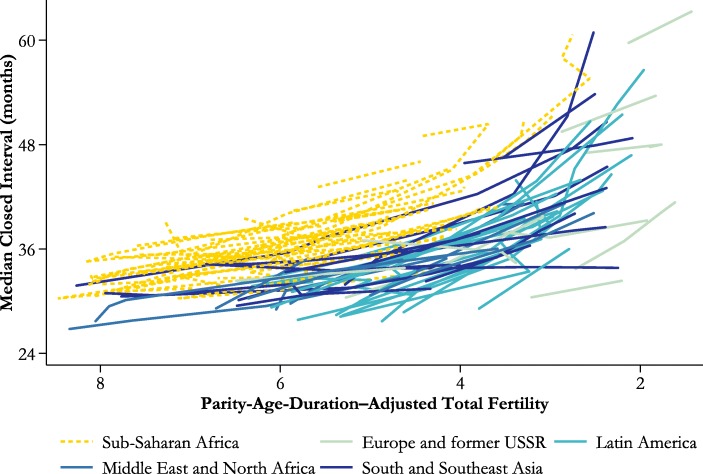
Trends in the median duration of closed birth intervals as total fertility decreases according to region in 83 countries

**Fig. 6:**
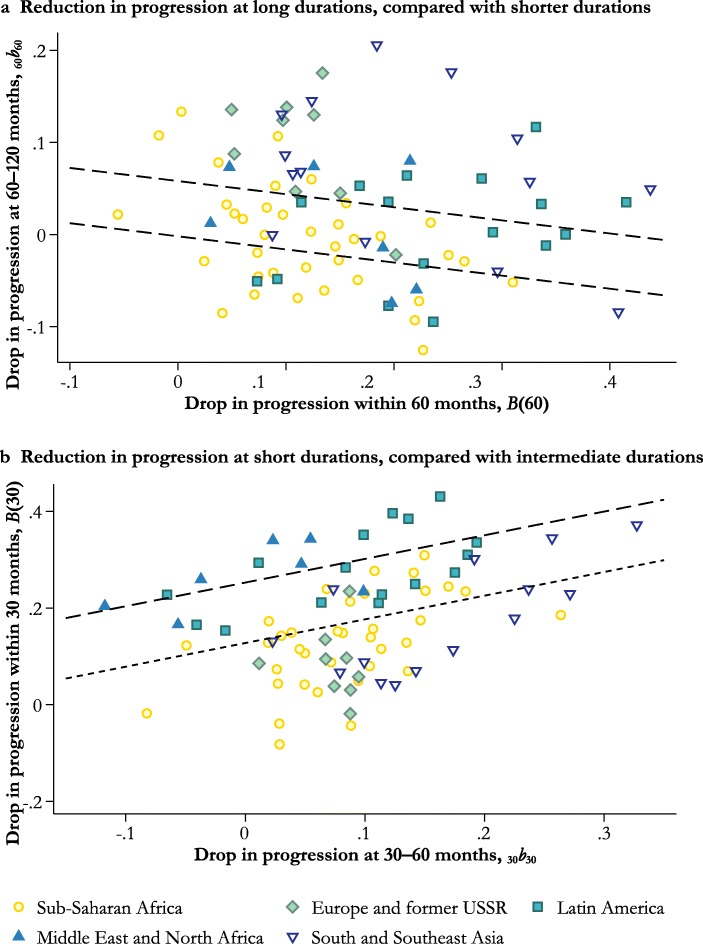
Reductions in the probabilities of progressing to the next birth according to the interval since the previous birth in 83 countries

Whereas panel a of Fig. 4 examines changes over time in patterns of parity progression, panel b summarizes the degree of curvature in the plots of the most recent estimates of progression to the next birth against parity. The higher of the dashed lines represents the contour at which the difference between PPR_4_ and PPR_8_ equals that between PPR_0_ and PPR_4_; countries above this line have convex curves. The lower line identifies countries in which the difference between PPR_0_ and PPR_4_ is at least three times the difference between PPR_4_ and PPR_8_; countries below this line have highly concave curves. Most African countries have convex curves or fairly straight ones. The two outlying African countries are Lesotho and South Africa. In contrast, most countries in other regions have concave or highly concave curves, with parity progression falling away rapidly between the first and fourth births. The exceptions are Afghanistan; Pakistan; the Middle East and North Africa (MENA) region, except Egypt and Turkey; Mexico prior to 1990; and East Timor. As in India and Kenya, plots of the proportion of women by parity who want no more children generally mirror those in parity progression. Thus, in countries in which the curves for parity progression have become highly concave, those for women wanting no more children are strongly convex; in countries where the former have remained more linear, so have the latter (see Fig. A4, online appendix).

Figure 5 presents trends in the period median duration of closed birth intervals. As total fertility fell, birth intervals lengthened in most countries and regions of the world. Median pretransition birth intervals were of the order of 30 months, a result consistent with the pioneering work of Sheps et al. (1973). They lengthened substantially after total fertility dropped below five children per woman. By the end of the study period, the median interval exceeded four years throughout Southern Africa; in Bangladesh, the Maldives, Myanmar, and Indonesia in Asia; in Colombia, Paraguay, and Peru in South America; and in Moldova and Ukraine in Europe. Thus, these synthetic cohort estimates for intervals of all orders agree broadly with those that Casterline and Odden (2016) made for the interval to the second birth.

Except in Southern Africa, the rate of increase in the median length of birth intervals in sub-Saharan Africa was similar to that in other regions. However, outside sub-Saharan Africa and the Caribbean, the latter region being one in which intervals were very short initially, the lengthening of birth intervals was concentrated in the second half of the fertility transition. Thus, controlling for total fertility, virtually no overlap exists between median birth intervals in African countries and those elsewhere.^7^ For example, outside sub-Saharan Africa, the only countries in which the median closed birth interval rose above three years before total fertility fell to fewer than 4.5 children per woman were Bangladesh and Tajikistan. In contrast, in every sub-Saharan African country in which total fertility dropped below 4.5 except Comoros, the median closed interval exceeded three years at that time.

A few countries saw no increase in the median length of closed birth intervals. The list includes several countries in the Sahel and Central Africa in which fertility changed little, but also Sierra Leone and Ethiopia, which are countries that saw a significant drop in total fertility. This group of countries also includes India (as shown in Fig. 3), Myanmar, Pakistan, and several countries in Central Asia.

Figure 6 examines the extent to which increases in the median length of birth intervals result from postponement and from birth spacing. Panel a looks at postponement. It compares changes in the period probability of having a birth 5–10 years after the previous birth, conditional on not having progressed before five years, with changes in the probability of progressing by five years since the previous birth. In countries above the upper dashed line, fertility fell by a relatively large amount at long durations, compared with medium and short durations. In countries below the lower dashed line, fertility rose over time at interval durations of 5–10 years, partly offsetting the drop in fertility that occurred at shorter durations.

The probability of progressing at birth intervals of 5–10 years is generally higher in sub-Saharan Africa than elsewhere. Moreover, many of the countries that saw progression at 5–10 years rise as progression before 5 years fell are in sub-Saharan Africa. In contrast, in most other countries, progression at 5–10 years fell along with progression before 5 years. The exceptions are Jordan, Morocco, Indonesia, Haiti, and some of the Latin American countries in which we could measure trends only prior to 1990. In many countries in which fertility at longer intervals fell, however, it did so relatively slowly, producing a flattening of the duration-specific fertility schedule. This flattening was more dramatic in sub-Saharan Africa than elsewhere.

Panel b of Fig. 6 presents an analogous analysis for birth spacing. It compares the period probability of closing the birth interval within 30 months of the previous birth with the probability of closing it in the following 30 months. The dotted line represents the regression of the first measure on the second one. Thus, we assess the trend toward *postponement* by comparing the standardized number of long intervals with the number of short and moderate ones, but we assess trends in *spacing* by comparing the standardized number of short intervals with the number of moderate length.

Trends in spacing have been muted in most countries. Short birth intervals became more common over time in about one-half the countries and less common in the others. In contrast to the previous figures, the cloud of points representing the sub-Saharan African countries largely overlaps with those for Europe and Asia. The dashed line of Fig. 6 identifies 11 countries in which a substantial reduction occurred in the proportion of closed intervals that were closed in less than 30 months. None of the 11 countries are in sub-Saharan Africa. Instead, they are confined to the MENA region, Latin America, and the Caribbean. The only countries that experienced increases in both postponement and spacing were Jordan, Morocco, and Costa Rica (where the run of data ends in 1990).

**Fig. 7:**
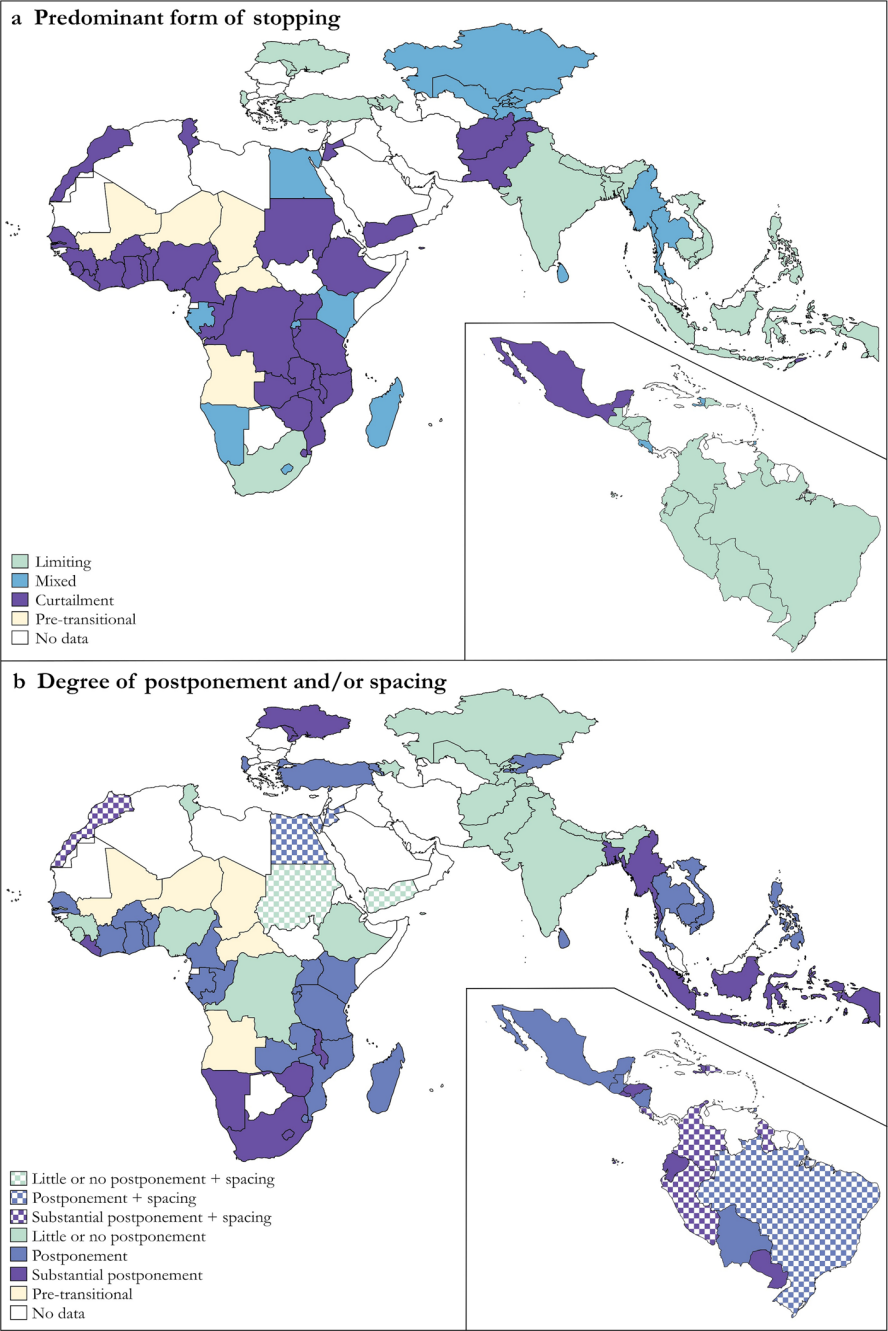
Pathways to low fertility, 1965–2014

Based on the results presented in Figs. 4–6 and Table 2, Table 3 presents a three-way classification of the pathways toward low fertility taken by 78 countries during the part of their transition documented by the surveys. Five African countries (Angola, Central African Republic, Chad, Mali, and Niger) in which the most recent estimate of total fertility exceeds six children were excluded from the table because fertility has not fallen enough to classify them. Figure 7 maps the countries according to this classification in order to draw out the geographical patterning of the results.

**Table 3:** Categorization of 78 countries according to the characteristics of their fertility transition

		Roles of Parity-Specific Limitation and Parity-Independent Curtailment		
Role of Spacing	Role of Postponement	Mostly Curtailment	Mixed	Mostly Limiting
Substantial increase in spacing	Substantial postponement	Morocco	Costa Rica	Colombia Dominican Republic Guyana Peru
	Postponement	Jordan	Egypt	Brazil
	Little or no postponement	Sudan (North) Yemen		
Limited or no increase in spacing	Substantial postponement	Malawi Liberia Zimbabwe	Lesotho Namibia Haiti Myanmar	South Africa Ecuador El Salvador Honduras Paraguay Moldova Ukraine Bangladesh Maldives Indonesia
	Postponement	Benin Burkina Faso Burundi Cameroon Congo (Republic) Côte d’Ivoire Ghana Mozambique São Tomé and Principe Senegal Swaziland Tanzania Togo Uganda Zambia Mexico	Trinidad and Tobago Gabon Kenya Madagascar Rwanda Kyrgyz Republic Sri Lanka Thailand	Bolivia Guatemala Nicaragua Albania Armenia Turkey Cambodia Philippines Vietnam
	Little or no postponement	Comoros Congo (Democratic Republic) Ethiopia Gambia Guinea Nigeria Sierra Leone Tunisia Afghanistan Pakistan Timor-Leste	Kazakhstan Tajikistan Uzbekistan	Azerbaijan India Nepal

Table 3 and panel a of Fig. 7 distinguish countries that have undergone a classic fertility transition, characterized by an increase in the proportion of women limiting childbearing to a small or moderate desired family size, from those in which stopping shows no evidence of being driven by parity-specific family size limitation. Countries that fall between these extremes are placed in a third, mixed category. The countries are divided horizontally in Table 3 and panel b of Fig. 7 into those characterized by the development of widespread and substantial postponement of the next birth, those with a smaller increase in postponement, and those in which no significant evidence of postponement exists. The upper of the two horizontal panels of Table 3 and hatched shading in panel b of Fig. 7 distinguish the 12 countries in which birth spacing increased substantially from those in which little change occurred or short birth intervals became more common.

Only a few countries followed a classic path to low fertility, characterized by parity-specific limitation with little increase in the length of birth intervals. This group is restricted to India and Nepal, together with Azerbaijan during the latter part of its transition. The only sub-Saharan African country in which family-size limitation predominated is South Africa. Although limitation is common outside sub-Saharan Africa, it is usually accompanied by increased postponement of the next birth, and in parts of Latin America, by birth spacing as well. Thus, in most countries characterized by parity-specific limitation, the median length of birth intervals has increased substantially.

In most of sub-Saharan Africa, in the Middle East and North African region, in Afghanistan, Pakistan, East Timor, and in Mexico prior to 1980, no evidence exists of family-size limitation. Instead, women reduced their number of births without converging on families of a particular size. Most of the African nations in this group of countries characterized by parity-independent curtailment of childbearing also experienced an increase in postponement. Most of its other members did not.

The intermediate group of countries that show evidence of a limited shift toward parity-specific limitation is fairly small. The countries are drawn from all regions of the world. They include the four Central Asian countries that were once part of the USSR and five mainland sub-Saharan African countries. With the exception of three of the Central Asian countries, this mixed group also saw an increase in postponement.

Fertility remained higher in most sub-Saharan African countries than in other regions at the end of the study period. This raises the possibility that postponement is not a geographically differentiated pattern of family building but rather a feature of the early phases of fertility transition. To assess whether this is the case, we repeated the classification of transitions into different pathways, examining only the initial drop in total fertility to four children per woman and discarding the more recent estimates (results not shown). The data on most African countries and the few other countries where the most recent total fertility rate exceeded four children were unaffected by this.

In most countries in which parity-specific limitation has played a part in the fertility transition, this pattern was already evident before total fertility fell to four children. Only a few countries experienced a period dominated by parity-independent curtailment followed by one dominated by parity-specific limitation. In addition to South Africa and the five relatively low fertility sub-Saharan African countries with a mixed pattern of parity progression at the end of the study period, these countries are Bangladesh, Egypt, and Indonesia. Turning to postponement, much of the lengthening of birth intervals in countries outside sub-Saharan Africa occurred during the second half of their fertility transition. Nevertheless, most countries characterized by substantial postponement at the end of the study period saw an initial shift in this direction before total fertility fell to four children per woman.

## Discussion

The concept of postponement originated in the discovery that very long birth intervals had emerged during the course of fertility transition in Southern Africa (Timæus and Moultrie 2008). It seemed implausible that birth spacing, as it is usually conceptualized, could produce such long intervals. Instead, increasing numbers of women were delaying their next birth for more than five years, suggesting that they were doing something different: postponing. This study adopts a wider perspective and examines both parity progression and birth interval dynamics by birth order. This approach has already proved informative in East Africa (Towriss and Timæus 2018) and becomes essential in analyses that extend beyond Africa to regions where parity-specific limitation is evidently important.

The first striking feature of the results presented here is the enormous variety in the pathways that different countries have taken through fertility transition. In some countries, fertility transition has been driven largely by a rise in the proportion of women who have exactly two children. In other countries, women have begun to restrict their family sizes without showing any sign of adopting small family norms. Instead, the more children a woman has had, the less likely she is to bear another. In many countries, birth intervals have also lengthened because of postponement, spacing, or both forms of birth control. In other countries, intervals have not changed at all. The pattern of fertility change tends to be similar in neighboring countries, but exceptions exist to almost every generalization about regional patterns of fertility decline that one might venture to make.

Our results confirm that fertility transition has been characterized by parity-specific limitation in most of the developing world outside sub-Saharan Africa. Yet, as Casterline and Odden (2016) pointed out, many countries outside Africa have also experienced some postponement of births and lengthening of birth intervals, especially in the second half of their fertility transitions. In contrast, the only evidence of parity-specific limitation in sub-Saharan Africa is in relatively low-fertility countries in Southern Africa, where it has only recently become apparent. Instead, parity progression in Africa has usually dropped the most at high parities, following a similar pattern to that shown for Kenya in Fig. 3. This produces either a steepening linear decline in progression with parity or a convex curve.

Birth intervals have lengthened in much of Africa, but did not do so everywhere. It was never our position that postponement is restricted to Africa. Rather, it is the importance of curtailment of childbearing and the unimportance of differences by parity, rather than the presence of postponement, that most clearly distinguish the first half of the fertility transition in sub-Saharan Africa from fertility transition in most of the rest of the world. Moreover, as we suggested in earlier work, in sub-Saharan Africa, “[B]irth intervals are largely independent of mother’s age and parity. By contrast, data from selected less developed countries in other regions, and from Europe early in its fertility transition, exhibit very different patterns” (Moultrie et al. 2012:253). Postponement and curtailment have emerged as relatively important drivers of fertility transition in sub-Saharan Africa not because postponement is restricted to Africa, but because, so far, parity-specific limitation of family size has been less prevalent in this region than elsewhere.

Although Africa is the region of the world in which postponement of the next birth until more than five years after the previous one is most prevalent, it is also among the world regions that have seen no increase in spacing, defined as a reduction in the proportion of closed birth intervals of less than 30 months. Only in a few countries globally has the entire fertility distribution shifted toward longer intervals over time. In general, little relationship exists between changes in the left and right tails of the fertility distribution. This represents further evidence that spacing and postponement are distinct phenomena, underlain by different sets of reasons for avoiding childbearing.

The results presented here provide few clues as to the institutional or cultural differences that underlie the diverse pathways toward low fertility that countries follow during their demographic transition. They do suggest, however, that women’s fertility intentions are closely interrelated with other aspects of their reproductive lives, including their relationships with men and their partner’s preferences. It is perhaps unsurprising that contraceptive sterilization is common only in countries in which limitation is the predominant form of birth control. However, perhaps the availability and promotion of contraceptive sterilization early in the fertility transition of countries such as India was one factor that encouraged the spread of parity-specific limitation. The relationship is clearly not a necessary one, though, because limitation predominates in those Latin American countries in which sterilization is relatively uncommon, such as Paraguay and Peru, as well as the larger number in which it is very common (UN Population Division 2018).

The acceptability of divorce and remarriage for women around the time fertility transition begins may also be a factor that helps to explain its course. Lengthy postponement of the next birth is common in sub-Saharan Africa, where rates of divorce and remarriage are generally fairly high (Clark and Brauner-Otto 2015), but is not found in India and other South Asian countries, where divorce and remarriage were almost unknown until recently. Within Asia, moreover, postponement is most prevalent in Indonesia, which is one of the few countries in the region in which marital instability has always been common (Dommaraju and Jones 2011).

The adoption of birth control to stop childbearing in the absence of parity-specific limitation at normative family sizes represents a challenge to existing demographic thinking about the process of fertility transition. The following quotations typify the dominant characterization of birth control in the field of demography:

[Birth] control can be said to exist when the behaviour of the couple is bound to the number of children already born and is modified when this number reaches the maximum which the couple does not want to exceed (Henry 1961:145).

Family limitation is deliberate restriction of the number of children born to couples who have reached a certain family size or parity (Pressat 1985:78).

Women who want to stop childbearing will be referred to as “limiters,” and those who have not yet achieved their desired family size as “spacers” (Bongaarts 1992:103).

Spacing behaviour refers to deliberate fertility control that is independent of parity (Okun 1995:86).

Neither Pressat’s (1985) definition of limitation nor Henry’s (1961) argument that limitation is evidence of volitional birth control is in itself problematic. What is problematic, however, is to invert Henry’s argument and assert that limitation is the only form of birth control just because it is impossible to determine whether other forms of birth control are being practiced using the historical data sets that interested Henry and were analyzed later for the European Fertility Project (Coale and Watkins 1986). This is widely recognized today. However, as exemplified by the second pair of quotations, although most demographers accept that limitation is not the only form of birth control, many of them claim, or take as axiomatic, that all birth control that is not limitation must be spacing. This claim is equally problematic.

It is difficult to see how the pattern of decline in parity progression documented here in most of Africa and the Middle East as well as in Afghanistan, Pakistan, and East Timor can result from women targeting a “certain” or “maximum” family size, to use the terms adopted by Pressat and Henry, respectively. Indeed, it is unclear from these data whether women are conceptualizing family size at all. Nevertheless, women in these populations are using birth control to stop childbearing, presumably because they want fewer children than they would have otherwise. Equally, we see no evidence of master schedule spacing—that is, the use of prolonged intervals to limit family size (Anderton and Bean 1985; Bongaarts and Potter 1983). Moreover, it seems perverse to claim that the drops in parity progression in these countries resulted from spacing. Not only does spacing related to the age of the youngest child have little impact on parity progression, but birth intervals have not lengthened *at all* in about one-third of the countries, including populous ones, such as Ethiopia, Nigeria, and Pakistan.

In some countries, the curtailment of childbearing may have spread because of a rise in proportion of women who are postponing their next birth and become perpetual postponers (Lightbourne 1985)—that is, women who never decide that now is the right time to have another baby. This is not the full story, however, because most of the countries outside Africa that are characterized by parity-independent curtailment—together with some sub-Saharan African countries, such as Ethiopia, Guinea, Nigeria, and Sierra Leone—show no evidence of an increase in postponement.

In a few of the countries, such as Ethiopia, fertility has fallen rather abruptly. In such countries, the pattern of decline in parity progression might result from the initial take-up, at a relatively late date in global terms, of birth control by a population that previously either lacked access to contraceptives or never realized that they could control their fertility. In other words, women started to limit their fertility at whatever family size they had reached at the time when contraception became available, and as younger cohorts build up their families, a more typical pattern of parity-specific limitation may arise. In most of the countries, however, fertility has declined too slowly for this account to be plausible. Instead, an increasing proportion of women may be using birth control, in effect, to retire from childbearing as they become older, more senior, and perhaps more concerned about their health; because their partner has deserted the family or they think that he might; or simply because they feel that they already have enough children to care for and educate (Agadjanian 2005; Bledsoe 2002; Garver 2018; Towriss et al. 2019).

The *curtailment* of childbearing, by which we mean a pattern of stopping childbearing that is independent of parity, is an important phenomenon that it is hard to reconcile with the traditional characterization of limitation. It seems unlikely, however, that a country could complete its transition to low fertility without curtailment giving way to parity-specific limitation. Low fertility requires most women to have no more than two children. Once women are having families that are this small, choices about whether to start and then how quickly to stop inevitably become issues of central importance to their reproductive lives. Thus, parity-specific limitation has played a role in the fertility decline in all the countries examined here in which total fertility is now less than four children per woman. Moreover, the five mainland sub-Saharan African countries classified as having a mixed pattern of decline in parity progression, together with South Africa, where limitation now predominates, are the six African countries in our study with the lowest fertility. It appears, however, that the argument that “a fertility decline is not very far away when people start conceptualizing their family size, and it cannot take place without such conceptualizing” (van de Walle 1992:502) may reverse the chain of events in at least some parts of the world. Curtailment occurs when people start to reduce the number of children they have without reference to a target family size—they just want fewer. It may be only as successive generations become increasingly confident of their ability to control their fertility that they start worrying about exactly how many children they do want.

The focus of this analysis is on aggregate fertility outcomes. It does not investigate individuals’ reported preferences and intentions beyond documenting that national-level trends in the proportion of women by parity who want no more children are broadly consistent with trends in parity progression. Nevertheless, the large and differential decreases in fertility over time documented in this article can result only from differential increases in volitional birth control rooted in varied changes in women’s fertility preferences and intentions.^8^ Our argument that women in many countries delay or stop childbearing for reasons other than family size limitation or spacing accords with evidence from both quantitative and qualitative research in several parts of Africa that has focused directly on women’s fertility preferences and intentions (Agadjanian 2005; Garver 2018; Hayford and Agadjanian 2017, 2019; Johnson-Hanks 2004; Towriss et al. 2019). Equally, the results presented here add heft to the work of anthropological demographers such as Johnson-Hanks, Bledsoe, and others who have argued that what women do in reality may be far removed from the oversimplified typifications adopted by many demographers and policy analysts (Bledsoe 2002; Johnson-Hanks 2005, 2007; Ware 1976).

The concepts of the curtailment and postponement of childbearing also align with recent literature emphasizing the uncertain, ambivalent, contingent, flexible, and fluid nature of the fertility intentions of women in both contemporary countries and historical Europe (Agadjanian 2005; Fisher 2000; Johnson-Hanks 2004, 2005; Ní Bhrolcháin and Beaujouan 2019; Towriss et al. 2019; Trinitapoli and Yeatman 2018; Yeatman et al. 2013). Limiters who have reached their desired family size might revisit their decision to stop childbearing in the (now rather uncommon) circumstance that one of their children dies. The considerations that lead women to curtail childbearing, however, may be both less clear-cut and more volatile. Similarly, women who are spacing will, in due course, either become pregnant or accomplish that aim. In contrast, women who are postponing childbearing for other reasons may never conclude that their situation has become more conducive to childbearing. Little ambiguity exists about when it is appropriate to limit or to space because decisions to do so are motivated by clearly defined demographic circumstances. The distinction between stopping childbearing and postponing the next birth is fuzzier when the decision has been motivated by factors that are largely unrelated to women’s reproductive histories (Hayford and Agadjanian 2019). Women who are avoiding childbearing for nondemographic reasons may not have decided, or even reflected on, whether they want another child later or not at all. Even if they have formulated their intentions, these may be tentative: the only decision that such women are impelled to make is whether to practice birth control at the current time (Ryder 1973).

One strength of the analysis in this article is that it integrates the regression modelling of period fertility using birth history data with a multistate life table model that calculates the PPRs and durations of birth intervals in a synthetic cohort that experiences the fertility rates of a specific period. Although this method of analysis has been proposed before (Rallu and Toulemon 1994; Retherford et al. 2013), nobody else has applied it previously to a large number of countries undergoing transition. The approach provides a more detailed description of the process of fertility transition across the developing world than has been available hitherto. As well as enabling us to examine progression and birth intervals by birth order, however, multistate modelling yields fully standardized estimates of trends in interval dynamics for all birth orders combined. In contrast, previous research has focused on the changes occurring in a particular birth interval or presented unstandardized measures for all intervals in which the distribution of births by order is determined by the history of fertility change in the population concerned, not by current conditions (e.g., Casterline and Odden 2016).

One limitation of this study is that the available fertility survey data often provide only a partial snapshot of the entire fertility transition in a country. In many Latin American and Asian countries, fertility transition was well underway a decade before they first conducted a fertility survey. Moreover, in most of sub-Saharan Africa, one can only speculate as to how family-building patterns may evolve during the second half of the fertility transition because this is yet to occur. Thus, our identification of the fertility transition in a country as characterized by limitation, curtailment, or postponement might require qualification if information existed on that country’s entire fertility transition. Nevertheless, analyzing the WFS data enables us to document the early stages of enough fertility transitions outside sub-Saharan Africa to make it clear that postponement is not a feature of the initial stages of fertility transition everywhere. Instead, outside sub-Saharan Africa, postponement generally becomes more prevalent as fertility falls to a low level. Parity-independent patterns of stopping, in contrast, seem destined to disappear as the fertility transition proceeds—or, rather, fertility will not fall to a low level until most women use birth control to stop childbearing when they have fewer than three children.

The (literal and conceptual) map that we draw of fertility transition across what was once termed “the developing world” is a complex one. Africa is not unique: a few other countries have experienced “African” transitions. Nevertheless, the overall picture is clear and spatially coherent. The initial stages of fertility transition in sub-Saharan Africa have followed a different track from that taken by almost all the rest of the world. The region has been characterized by the curtailment and (in most countries) postponement of childbearing, without the development of clear-cut preferences for small desired family sizes. The pace of fertility decline in Africa will remain slow until large numbers of African women start limiting their families to only a few children. Gaining a better understanding of the *motivations* that underlie African women’s family building patterns is essential for the development of appropriate reproductive health care services for Africa. Gaining a better understanding of the *consequences* of those patterns is vital if we are to understand their implications for future fertility and population growth, not just in sub-Saharan Africa, but globally.

Developing typologies is ultimately an arid exercise if it fails to point the way to explanations. The pathways through fertility transition documented here suggest that in many countries, rather than having a master schedule for their reproductive lives, most women plan their families as they go. If they have enduring quantitative fertility preferences, these are probably numerically imprecise, such as “at least two” or “fewer than my mother.” Wanting to have a(nother) child now, later, or not at all, together with being unsure whether one wants a child later, are an exhaustive and mutually exclusive set of possibilities. Parity-specific family size limitation and birth spacing are not. The terms usefully encapsulate the two main motivations for practicing birth control that relate to women’s reproductive histories. However, characterizing all birth control as either limitation or spacing systematically diverts attention away from nondemographic reasons for intentionally stopping childbearing or postponing having another birth. Changes across the less-developed world during the last half-century in patterns of parity progression by birth order, the length of birth intervals, and interval-duration–specific fertility demonstrate that limitation and spacing are not the only important motivations for adopting birth control. In many countries, large numbers of women practice birth control to stop childbearing for reasons other than limiting their families to some desired size or to postpone having another birth for reasons unrelated to the age of their youngest child.

## Electronic supplementary material

ESM 1 (PDF 345 kb)

## Footnotes

1 We thank Simon Szreter for suggesting *curtailment* as a concise term for “parity-independent stopping.”

2 Defining short intervals in other ways—for example, as intervals of less than 24 months—produces very similar results.

3 Of course, typically births are spaced out by other means even before the fertility transition begins (e.g., Fisher and Szreter 2003).

4 In the interests of reproducibility, the model coefficients and derived indicators, together with the Stata *do* files and Excel spreadsheets used to undertake the analysis and produce the figures in this article, are available via its webpage (https://bugbunny.github.io/Pathways_to_Low_Fertility/) and on *GitHub* (https://github.com/BugBunny/Pathways_to_Low_Fertility).

5 Corresponding figures for all 83 countries are presented in Figs. A1–A4 of the online appendix.

6 Most of India has followed broadly the same pathway through fertility transition, albeit timed differently. When the country is divided into six zones, the only substantial regional difference that is evident is that parity-specific limitation has been accompanied by increased postponement of births in the northeastern states of the country (results not shown).

7 The Asian country with birth intervals that are as long as, and have lengthened like, those in an “African” country, according to Fig. 5, is Bangladesh.

8 This statement does not imply that we accept a rational choice model of fertility decision-making.
