# Supplementary material for: Pathways to Low Fertility: 50 Years of Limitation, Curtailment, and Postponement of Childbearing
Source: Demography. 2020 Jan 22;57(1):267–96. doi: 10.1007/s13524-019-00848-5 (PMC7051933; doi:10.1007/s13524-019-00848-5)
Supplement: Supplementary file 1 — (PDF 345 kb) [file 13524_2019_848_MOESM1_ESM.pdf]

Online Appendix
to
Pathways to Low Fertility: 50 Years of Limitation, Curtailment, and Postponement of Childbearing

(Published in *Demography*, 57 (2020). DOI: 10.1007/s13524-019-00848-5)

Ian M. Timæus and Tom A. Moultrie

Table A1 Countries and surveys included in the analysis

Country		Region	Sub-region	Surveys								
Afghanistan	AF	South & Southeast Asia	South Asia	2015								
Albania	AL	Europe & former USSR		2008-2009	2017-2018							
Angola	AO	Africa	Middle Africa	2015-2016								
Armenia	AM	Europe & former USSR		2000	2005	2010	2015-2016					
Azerbaijan	AZ	Europe & former USSR		2006								
Bangladesh	BD	South & Southeast Asia	South Asia	1975*	1993-1994	1996-1997	1999-2000	2004	2007	2011	2014	
Benin	BJ	Africa	West Africa	1981*	1996	2001	2006	2011-2012				
Bolivia	BO	Latin America	South America	1989	1994	1998	2003	2008				
Brazil	BR	Latin America	South America	1986	1991	1996						
Burkina Faso	BF	Africa	West Africa	1993	1998-1999	2003	2010					
Burundi	BU	Africa	East Africa	1987	2010	2016-2017						
Cambodia	KH	South & Southeast Asia	Southeast Asia	2000	2005	2010	2014					
Cameroon	CM	Africa	Middle Africa	1978*	1991	1998	2004	2011				
Central African Rep.	CF	Africa	Middle Africa	1994-1995								
Chad	TD	Africa	Middle Africa	1996-1997	2004	2014-2015						
Colombia	CO	Latin America	South America	1976*	1986	1990	1995	2000	2005	2010	2015	
Comoros	KM	Africa	East Africa	1996	2012							
Congo (DRC)	CD	Africa	Middle Africa	2007	2013-2014							
Congo (Republic)	CG	Africa	Middle Africa	2005	2011-2012							
Costa Rica	CR	Latin America	Caribbean	1976*	1993**							
Côte d'Ivoire	CI	Africa	West Africa	1980*	1994	1998-1999	2011-2012					
Dominican Republic	DR	Latin America	Caribbean	1975*	1980*	1986	1991	1996	1999	2002	2007	2013
Ecuador	EC	Latin America	South America	1979*	1987	1994**	1999**	2004**				
Egypt	EG	Middle East & N. Africa		1980*	1988	1992	1995	2000	2005	2008	2014	
El Salvador	ES	Latin America	Caribbean	1985	1998**	2003**	2008**					

Country		Region	Sub-region	Surveys							
Ethiopia	ET	Africa	East Africa	2000	2005	2011	2016				
Gabon	GA	Africa	Middle Africa	2000	2012						
The Gambia	GM	Africa	West Africa	2013							
Ghana	GH	Africa	West Africa	1979*	1988	1993	1998	2003	2008	2014	
Guatemala	GU	Latin America	Caribbean	1987	1995	2002**	2008**	2014-2015			
Guinea	GN	Africa	West Africa	1999	2005	2012					
Guyana	GY	Latin America	South America	1975*	2009						
Haiti	HT	Latin America	Caribbean	1977*	1994-1995	2000	2005-2006	2012	2016-2017		
Honduras	HN	Latin America	Caribbean	1996**	2001**	2005-2006	2011-2012				
India	IA	South & Southeast Asia	South Asia	1992-1993	1998-1999	2005-2006	2015-2016				
Indonesia	ID	South & Southeast Asia	Southeast Asia	1976*	1987	1991	1994	1997	2002-2003	2007	2012
Jordan	JO	Middle East & N. Africa		1976*	1990	1997	2002	2007	2012		
Kazakhstan	KK	Europe & former USSR		1995	1999						
Kenya	KE	Africa	East Africa	1977*	1989	1993	1998	2003	2008-2009	2014	
Kyrgyz Republic	KY	Europe & former USSR		1997	2012						
Lesotho	LS	Africa	Southern Africa	1977*	2004	2009	2014				
Liberia	LB	Africa	West Africa	1986	2007	2013					
Madagascar	MD	Africa	East Africa	1992	1997	2003-2004	2008-2009				
Malawi	MW	Africa	East Africa	1992	2000	2004	2010	2015-2016			
Maldives	MV	South & Southeast Asia	South Asia	2009							
Mali	ML	Africa	West Africa	1987	1995-1996	2001	2006	2012-2013			
Mexico	MX	Latin America	Caribbean	1976*	1987						
Moldova	MB	Europe & former USSR		2005							
Morocco	MA	Middle East & N. Africa		1980*	1987	1992	2003-2004				
Mozambique	MZ	Africa	East Africa	1997	2003	2011					
Myanmar	MM	South & Southeast Asia	Southeast Asia	2015-2016							
Namibia	NM	Africa	Southern Africa	1992	2000	2006-2007	2013				
Nepal	NP	South & Southeast Asia	South Asia	1976*	1996	2001	2006	2011	2016		
Nicaragua	NC	Latin America	Caribbean	1992**	1998	2001	2006**				
Niger	NI	Africa	West Africa	1992	1998	2006	2012				
Nigeria	NG	Africa	West Africa	1981*	1990	1999	2003	2008	2013		
Pakistan	PK	South & Southeast Asia	South Asia	1975*	1990-1991	2006-2007	2012-2013				

**Country**		**Region**	**Sub-region**	**Surveys**						
Paraguay	PY	Latin America	South America	1979*	1990	1995**	1998**	2004**	2008**	
Peru	PE	Latin America	South America	1977*	1991-1992	1996	2000	2004-2006†	2009-2012†	
Philippines	PH	South & Southeast Asia	Southeast Asia	1978*	1993	1998	2003	2008	2013	2017
Rwanda	RW	Africa	East Africa	1983*	1992	2000	2005	2010	2014-15	
São Tomé & Principe	ST	Africa	Middle Africa	2008-2009						
Senegal	SN	Africa	West Africa	1978*	1986	1992-1993	1997	2005	2010-2011	2012-2017†
Sierra Leone	SL	Africa	West Africa	2008	2013					
South Africa	ZA	Africa	Southern Africa	1998	2016					
Sri Lanka	LK	South & Southeast Asia	South Asia	1975*	1987					
Sudan	SD	Middle East & N. Africa		1978*	1989-1990					
Swaziland	SZ	Africa	Southern Africa	2006-2007						
Tajikistan	TJ	Europe & former USSR		2012	2017					
Tanzania	TZ	Africa	East Africa	1991-1992	1996	1999	2004-2005	2010	2015-2016	
Thailand	TH	South & Southeast Asia	Southeast Asia	1975*	1987					
Timor-Leste	TL	South & Southeast Asia	Southeast Asia	2009-2010	2016					
Togo	TG	Africa	West Africa	1988	1998	2013-2014				
Trinidad & Tobago	TT	Latin America	Caribbean	1977*	1987					
Tunisia	TN	Middle East & N. Africa		1978*	1988					
Turkey	TR	Middle East & N. Africa		1975*	1993	1998	2003			
Uganda	UG	Africa	East Africa	1988-1989	1995	2000-2001	2006	2011	2016	
Ukraine	UA	Europe & former USSR		2007						
Uzbekistan	UZ	Europe & former USSR		1996						
Vietnam	VN	South & Southeast Asia	Southeast Asia	1997	2002					
Yemen	YE	Middle East & N. Africa		1979*	1991-1992	2013				
Zambia	ZM	Africa	East Africa	1992	1996	2001-2002	2007	2013-2014		
Zimbabwe	ZW	Africa	East Africa	1988	1994	1999	2005-2006	2010-2011	2015	

*World Fertility Survey **Reproductive Health Survey (all other surveys are Demographic and Health Surveys). † Continuous DHS

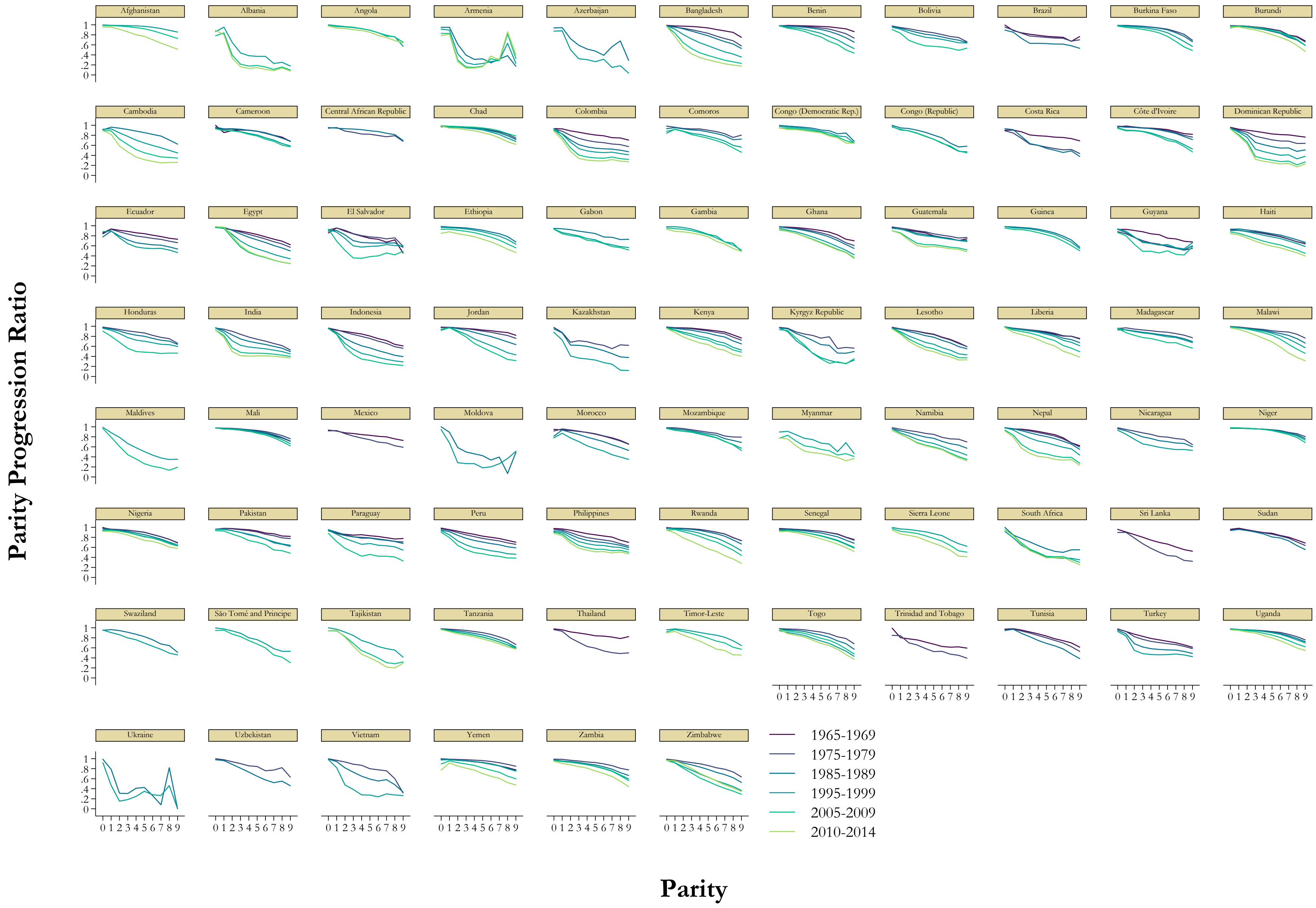

Fig. A1 Progression ratios according to women's parity by quinquennium in 83 countries

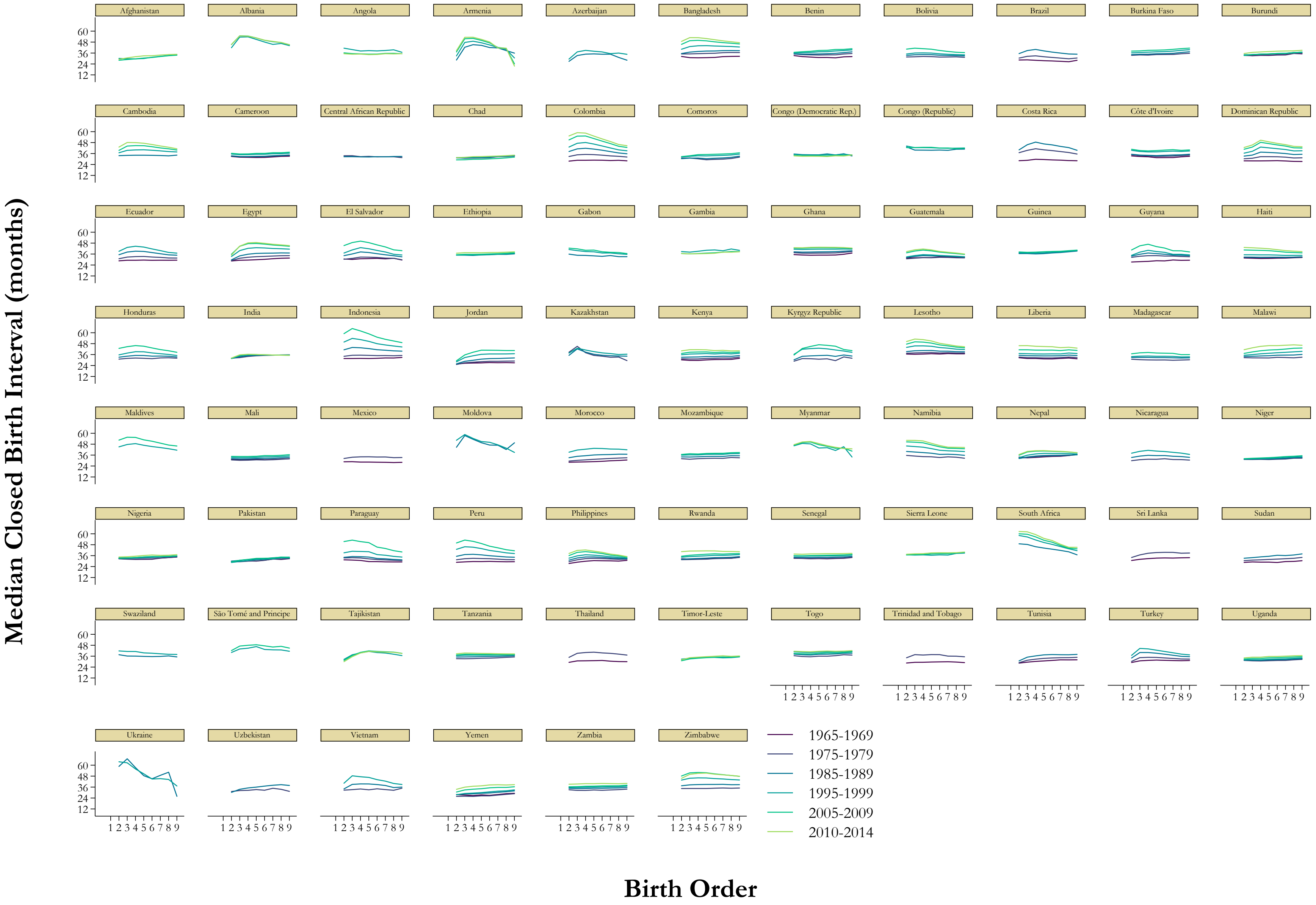

Fig. A2 Median duration of closed birth intervals according to women's parity by quinquennium in 83 countries

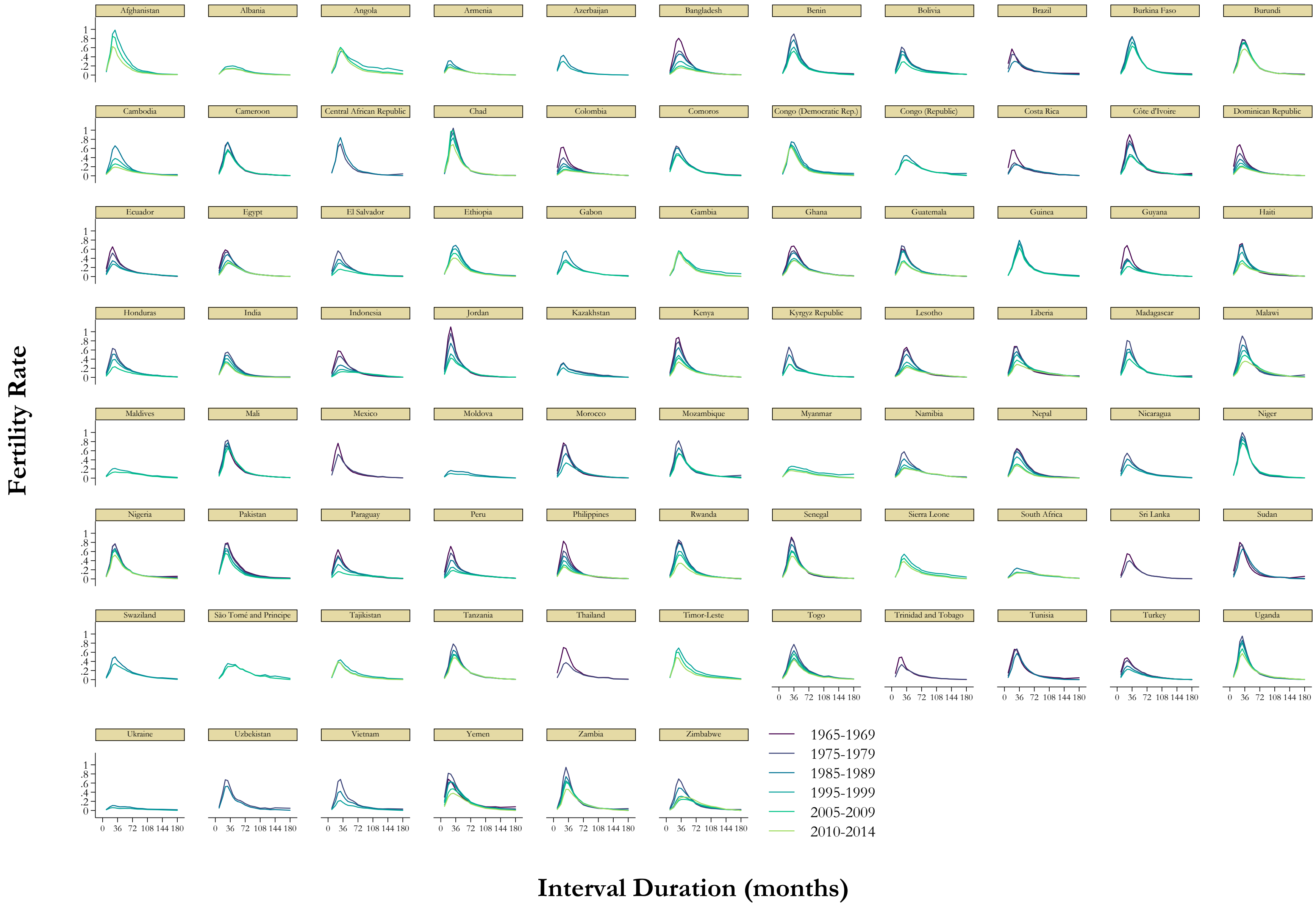

Fig. A3 Duration-specific fertility rates by quinquennium in 83 countries

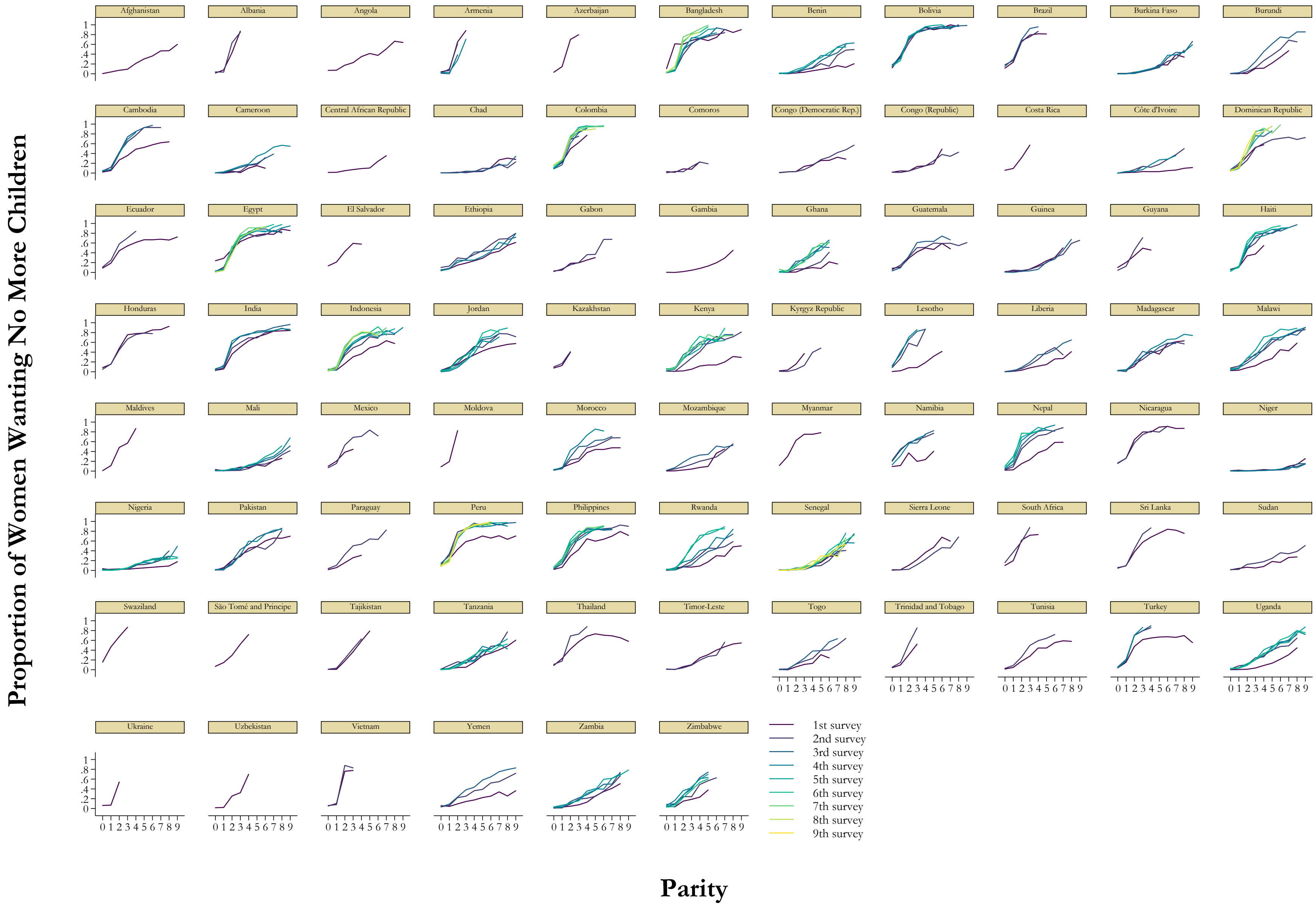

Fig. A4 Proportions of fertile married women who gave birth in the previous year or were childless reporting that they want no more children according to women's parity by survey in 83 countries (Proportions based on fewer than 50 respondents have been suppressed)
